# Supplementary material for: In Vitro Microevolution and Co-Selection Assessment of Amoxicillin and Cefotaxime Impact on Escherichia coli Resistance Development
Source: Antibiotics (Basel). 2024 Mar 7;13(3):247. doi: 10.3390/antibiotics13030247 (PMC10967516; doi:10.3390/antibiotics13030247)
Supplement: Supplementary file 1 [file antibiotics-13-00247-s001.zip › File S1. Supplementary materials.zip!/Supplementary materials.pdf]

Supplementary Table S1: Quality data of the contigs based on the QUAST (v5.2.) software analysis.

Strain	No of trimmed reads	No of contigs	Coverage	N50	N75	L50	L75
0× AMX	4956318	109	180.18	231989	121408	6	14
1× AMX	6628030	111	259.102	205034	121530	7	14
10× AMX	4791998	126	168.046	194885	107365	9	18
100× AMX	5440885	193	134.166	160547	84576	12	24
1000× AMX	4221108	102	161.305	202436	130873	8	16
0× CTX	2277416	249	55.8348	120 956	65 648	14	27
1× CTX	2421900	253	59.5407	132 252	57 752	14	27
10× CTX	2444649	237	59.7927	155 433	84 590	12	24
100× CTX	2315672	260	56.5416	120 953	50 443	15	29
1000× CTX	1413575	327	34.4063	73 430	29 355	21	48

AMX-amoxicillin, CTX-cefotaxime

Supplementary Table S2: The set of 44 ARGs identified in the next-generation sequencing was the same for all samples.

Gene	Coverage %	Identity %	Mechanism	Resistance
acrA	100.00	99.16	antibiotic efflux	cephalosporin, fluoroquinolone, glycylcycline, penam, phenicol, rifamycin, tetracycline and triclosan
acrB	100.00	98.64	antibiotic efflux	
acrD	100.00	98.04	antibiotic efflux	aminoglycoside
acrE	100.00	98.79	antibiotic efflux	cephalosporin, cephamycin, fluoroquinolone and penam
acrF	100.00	96.49	antibiotic efflux	
acrS	100.00	98.34	antibiotic efflux	cephalosporin, cephamycin, fluoroquinolone, glycylcycline, penam, phenicol, rifamycin, tetracycline and triclosan
ampC	100.00	98.15	antibiotic inactivation	cephalosporin and penam
ampH	100.00	97.50	antibiotic inactivation	
bacA	99.76	98.17	target alteration	peptide
baeR	99.86	96.81	antibiotic efflux	aminocoumarin andaminoglycoside
baeS	100.00	90.53	antibiotic efflux	
cpxA	100.00	98.47	antibiotic efflux	
CRP	100.00	99.21	antibiotic efflux	fluoroquinolone, macrolide and penam
emrA	100.00	98.21	antibiotic efflux	fluoroquinolone
emrB	100.00	96.95	antibiotic efflux	
emrE	100.00	92.19	antibiotic efflux	macrolide
emrK	100.00	97.73	antibiotic efflux	tetracycline
emrR	100.00	98.68	antibiotic efflux	fluoroquinolone
emrY	100.00	97.73	antibiotic efflux	tetracycline
eptA	100.00	91.85	target alteration	peptide
evgA	100.00	99.02	antibiotic efflux	fluoroquinolone, macrolide, penam and tetracycline
evgS	100.00	96.19	antibiotic efflux	
gadW	100.00	99.86	antibiotic efflux	fluoroquinolone, macrolide and penam
gadX	100.00	93.82	antibiotic efflux	

H-NS	100.00	99.28	antibiotic efflux	cephalosporin, cephamycin, fluoroquinolone, macrolide, penam and tetracycline
kdpE	99.26	95.84	antibiotic efflux	aminoglycoside
marA	100.00	98.70	reduced permeability	carbapenem, cephalosporin, cephamycin, fluoroquinolone, glycylcycline, monobactam, penam, penem, phenicol, rifamycin, tetracycline and triclosan
mdfA	100.00	96.59	antibiotic efflux	benzalkonium chloride, rhodamine and tetracycline
mdtA	100.00	95.11	antibiotic efflux	aminocoumarin
mdtB	100.00	96.29	antibiotic efflux	
mdtC	100.00	94.15	antibiotic efflux	
mdtE	100.00	98.62	antibiotic efflux	fluoroquinolone, macrolide and penam
mdtF	100.00	97.33	antibiotic efflux	
mdtG	100.00	98.21	antibiotic efflux	fosfomycin
mdtH	100.00	98.26	antibiotic efflux	fluoroquinolone
mdtM	100.00	95.05	antibiotic efflux	acridine dye, fluoroquinolone, lincosamide, nucleoside and phenicol
mdtN	100.00	95.64	antibiotic efflux	acridine dye and nucleoside
mdtO	100.00	97.08	antibiotic efflux	
mdtP	100.00	97.61	antibiotic efflux	
msbA	100.00	98.06	antibiotic efflux	nitroimidazole
pmrF	100.00	97.63	target alteration	peptide
tolC	100.00	97.98	antibiotic efflux	aminocoumarin, aminoglycoside, carbapenem, cephalosporin, cephamycin, fluoroquinolone, glycylcycline, macrolide, penam, penem, peptide, phenicol, rifamycin, tetracycline and triclosan
ugd	100.00	96.92	target alteration	peptide
yoiI	100.00	98.05	antibiotic efflux	

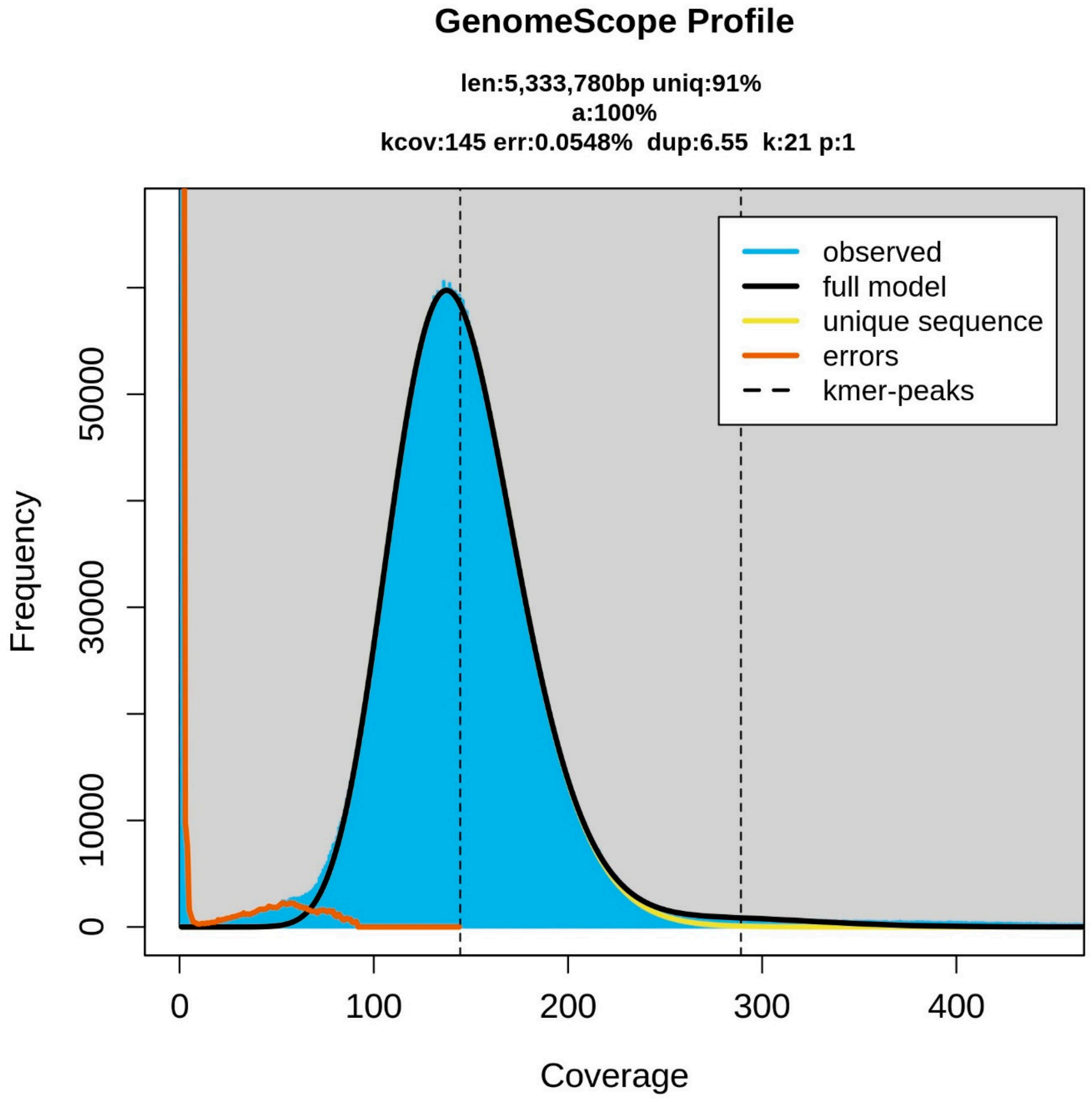

Supplementary Figure S1: GenoScope profile of 0× AMX sample. The fit of the model (black line) to the observed kmer fre-quencies (blue area) is shown for E. coli sample. The genome size, heterozygote ratio and repetitive content of unprocessed short reads can be read.

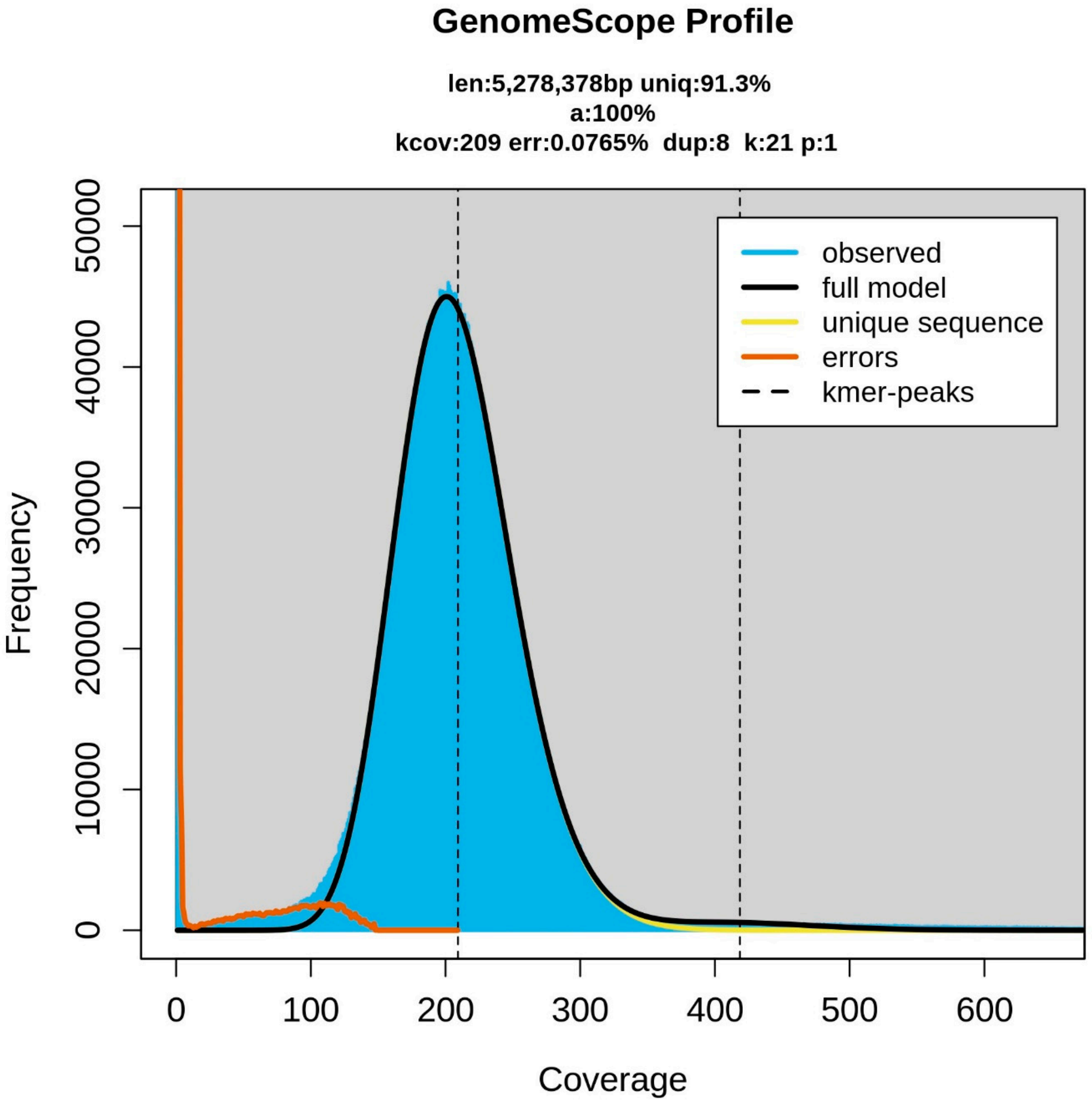

Supplementary Figure S2: GenoScope profile of 1× AMX sample. The fit of the model (black line) to the observed kmer fre-quencies (blue area) is shown for E. coli sample. The genome size, heterozygote ratio and repetitive content of unprocessed short reads can be read.

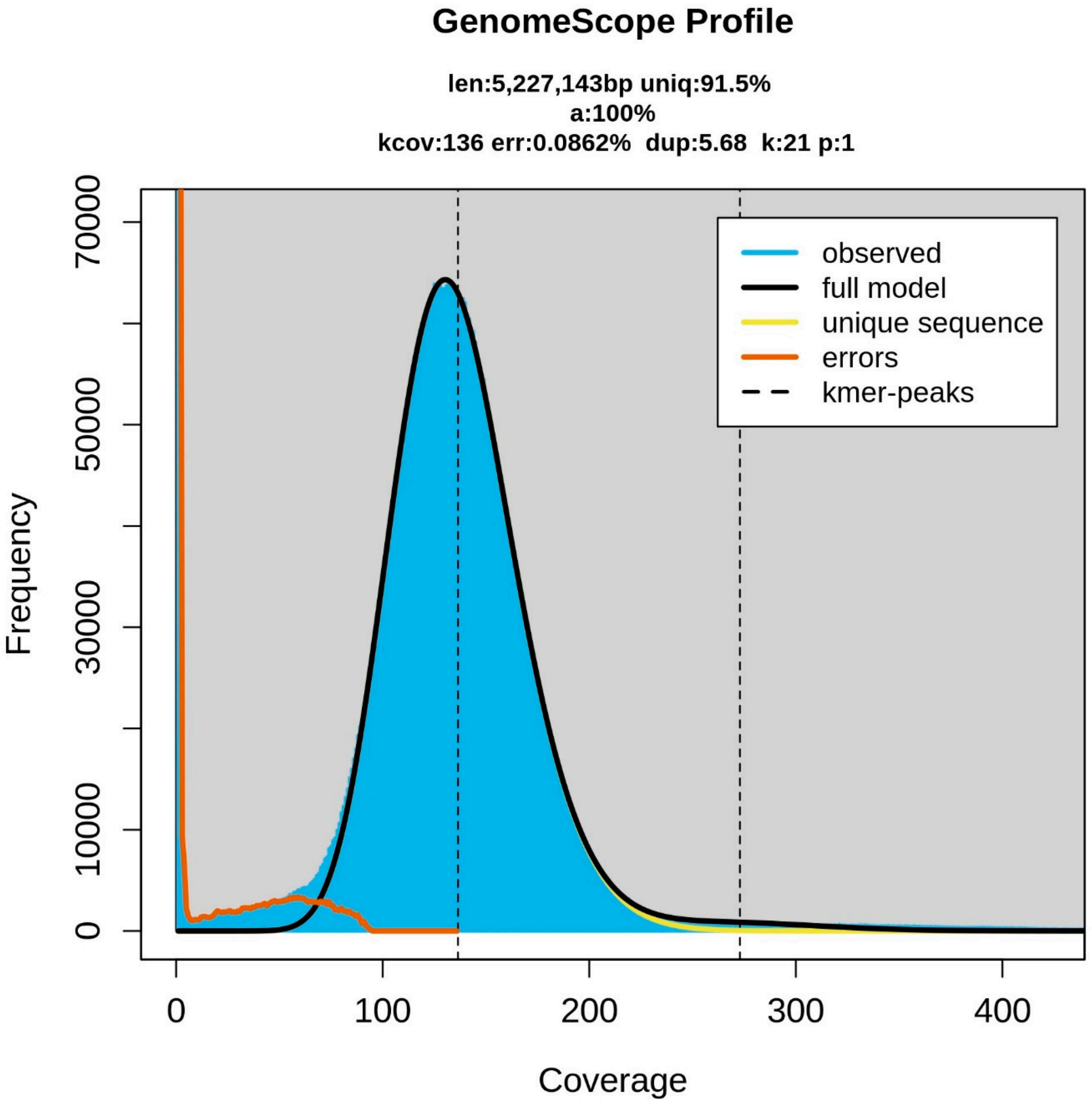

Supplementary Figure S3: GenoScope profile of 10× AMX sample. The fit of the model (black line) to the observed kmer fre-quencies (blue area) is shown for E. coli sample. The genome size, heterozygote ratio and repetitive content of unprocessed short reads can be read.

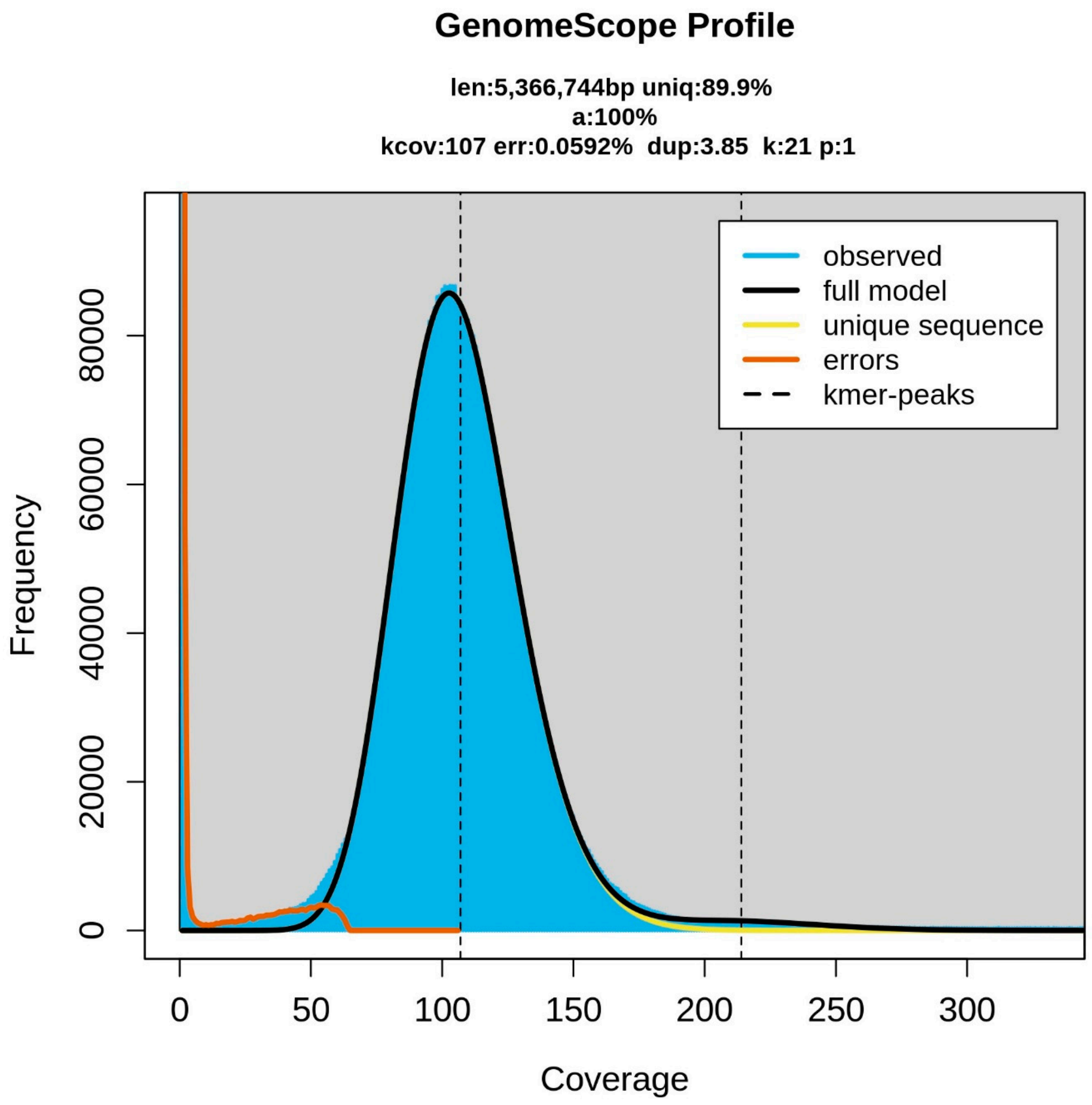

Supplementary Figure S4: GenoScope profile of 100× AMX sample. The fit of the model (black line) to the observed kmer fre-quencies (blue area) is shown for E. coli sample. The genome size, heterozygote ratio and repetitive content of unprocessed short reads can be read.

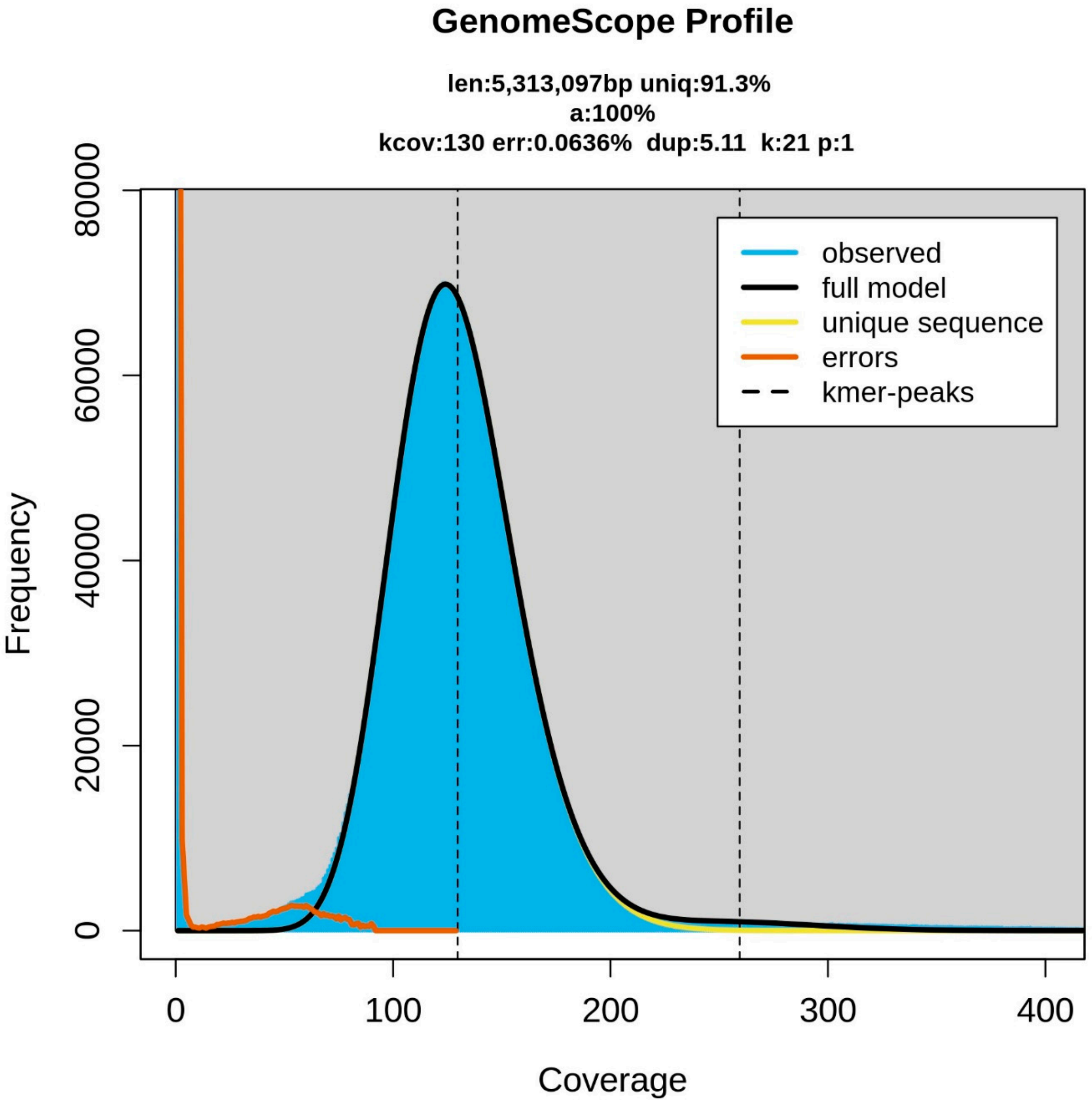

Supplementary Figure S5: GenoScope profile of 1000× AMX sample. The fit of the model (black line) to the observed kmer fre-quencies (blue area) is shown for E. coli sample. The genome size, heterozygote ratio and repetitive content of unprocessed short reads can be read.

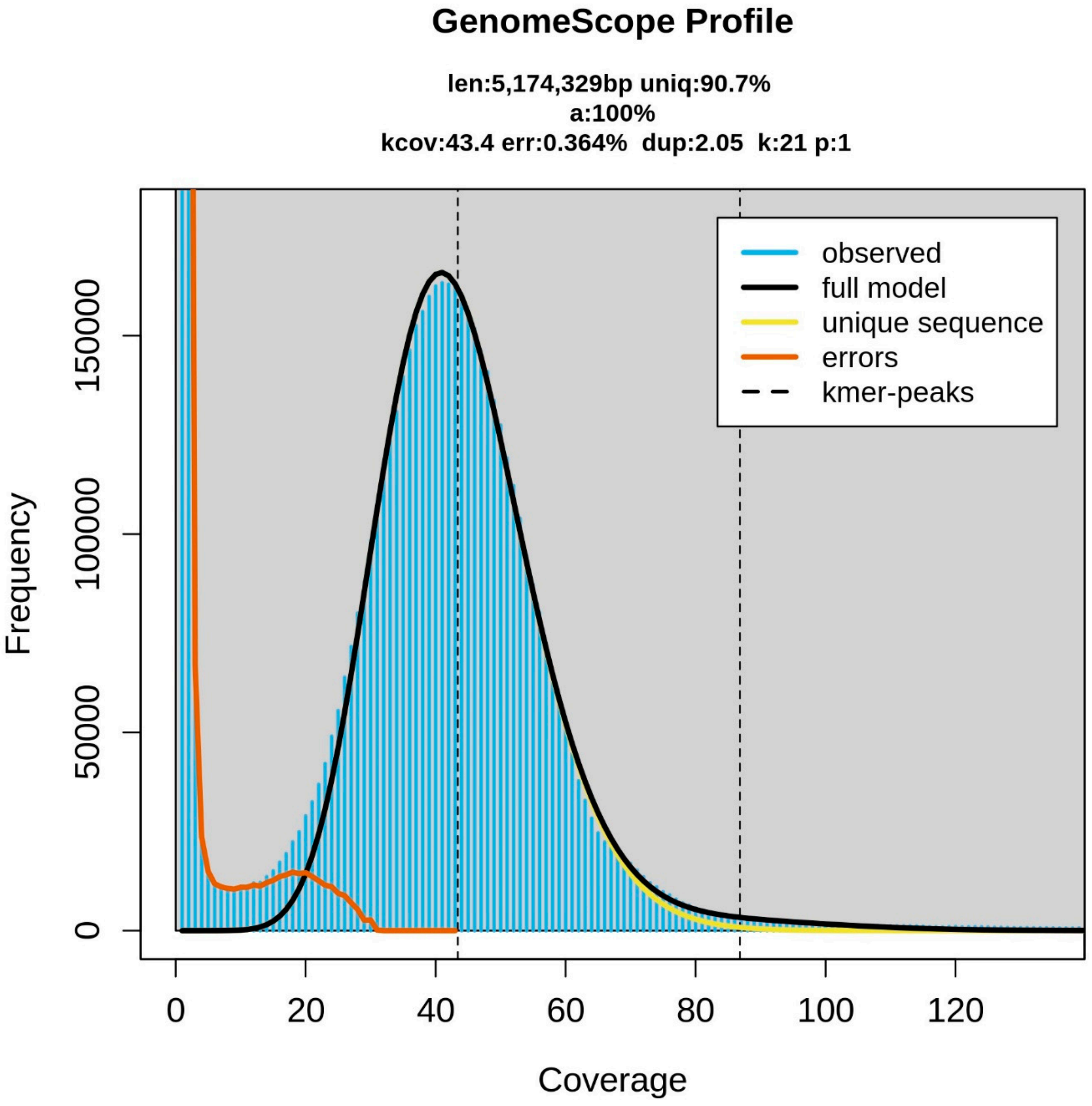

Supplementary Figure S6: GenoScope profile of 0× CTX sample. The fit of the model (black line) to the observed kmer fre-quencies (blue area) is shown for E. coli sample. The genome size, heterozygote ratio and repetitive content of unprocessed short reads can be read.

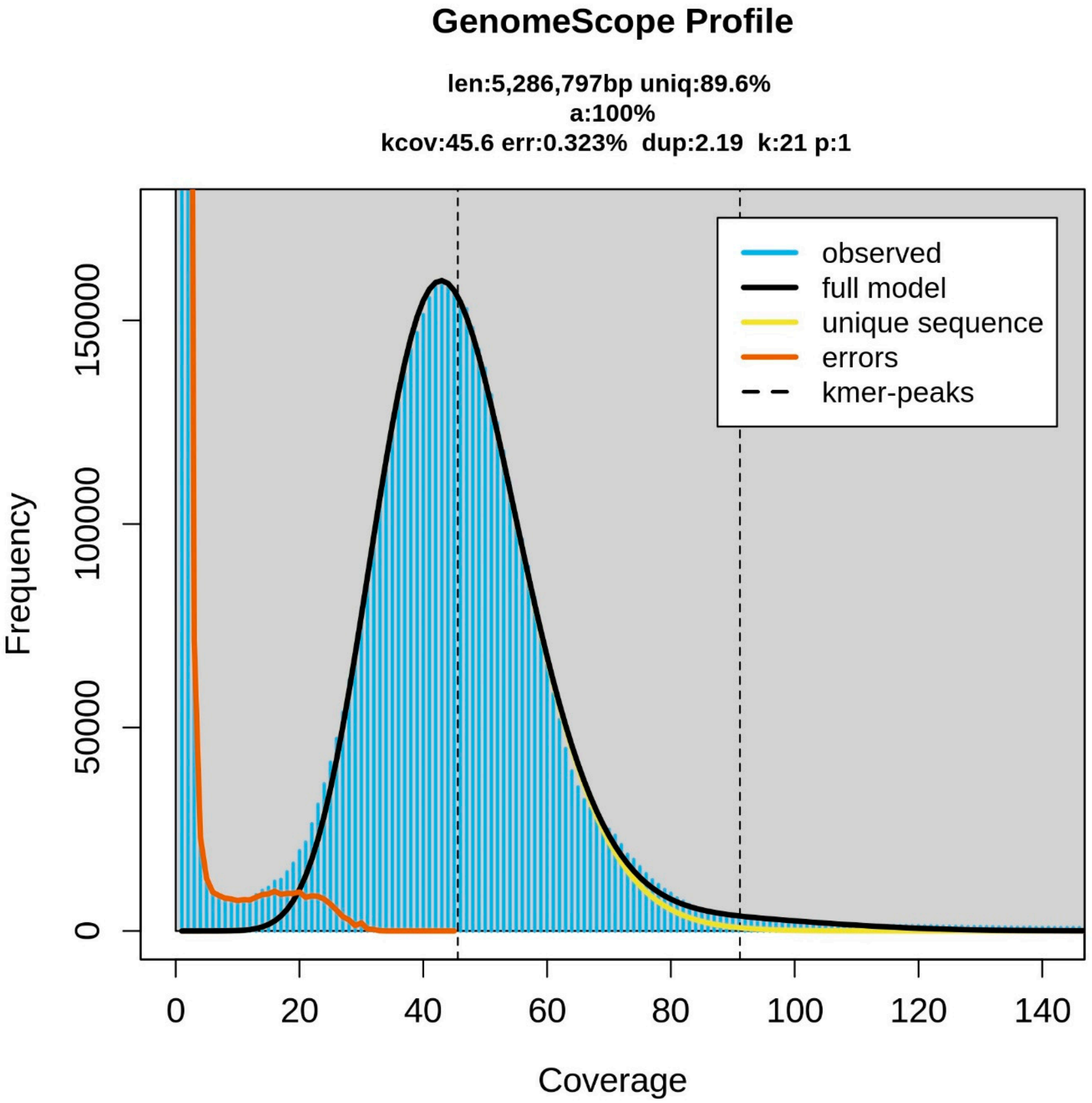

Supplementary Figure S7: GenoScope profile of 1× AMX sample. The fit of the model (black line) to the observed kmer fre-quencies (blue area) is shown for E. coli sample. The genome size, heterozygote ratio and repetitive content of unprocessed short reads can be read.

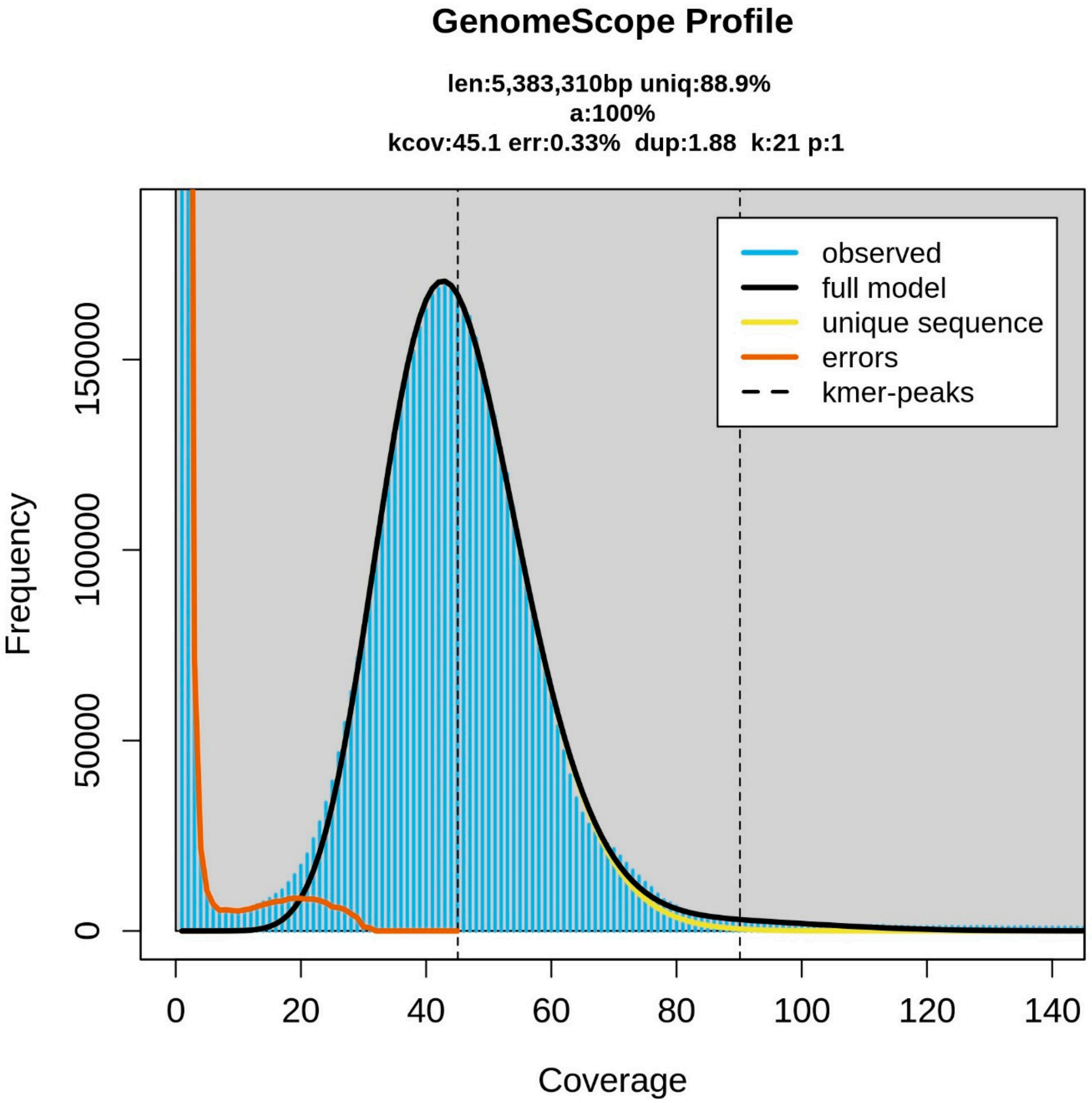

Supplementary Figure S8: GenoScope profile of 10× AMX sample. The fit of the model (black line) to the observed kmer fre-quencies (blue area) is shown for E. coli sample. The genome size, heterozygote ratio and repetitive content of unprocessed short reads can be read.

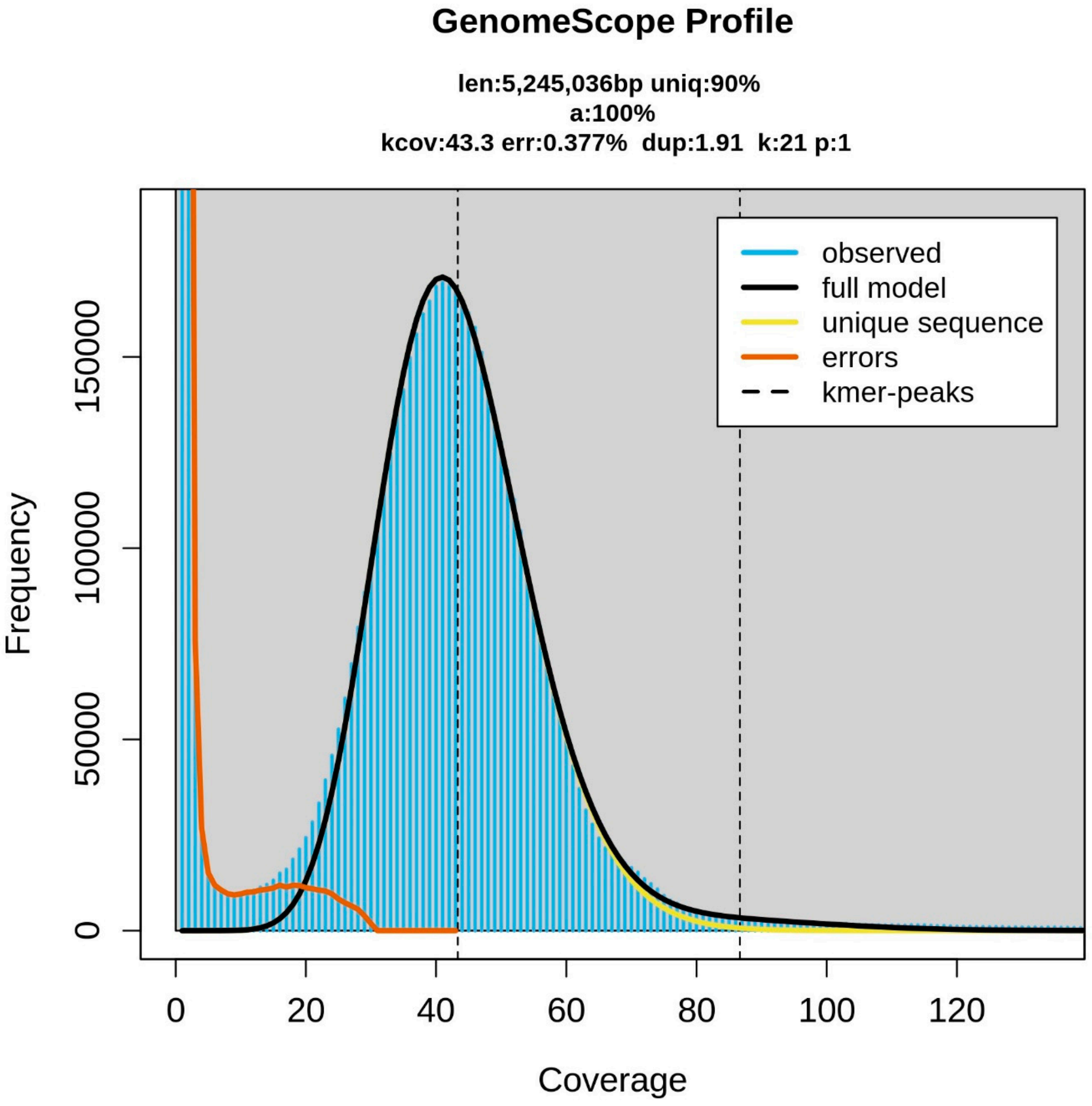

Supplementary Figure S9: GenoScope profile of 100× AMX sample. The fit of the model (black line) to the observed kmer fre-quencies (blue area) is shown for E. coli sample. The genome size, heterozygote ratio and repetitive content of unprocessed short reads can be read.

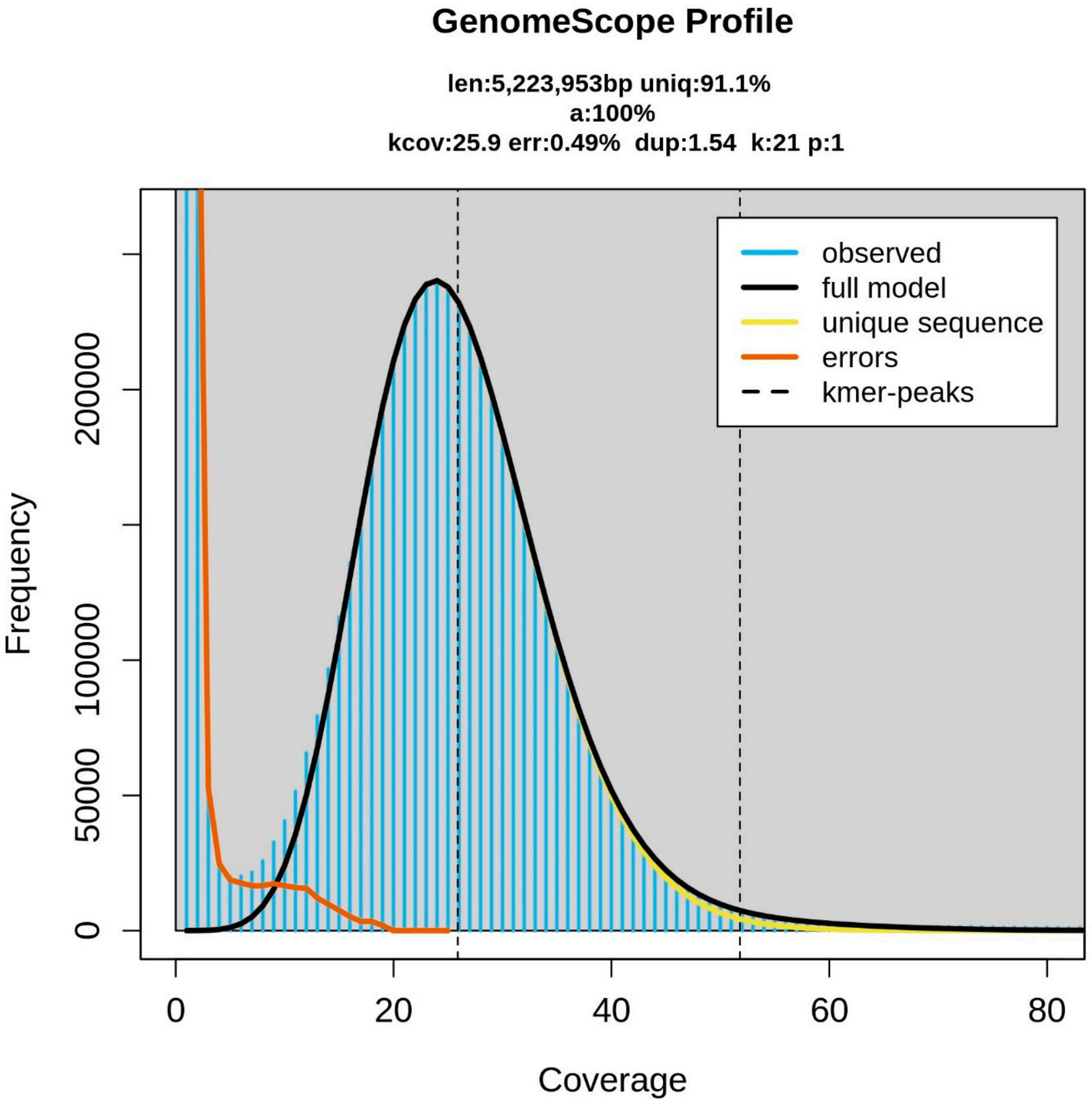

Supplementary Figure S10: GenoScope profile of 1000× AMX sample. The fit of the model (black line) to the observed kmer fre-quencies (blue area) is shown for E. coli sample. The genome size, heterozygote ratio and repetitive content of unprocessed short reads can be read.

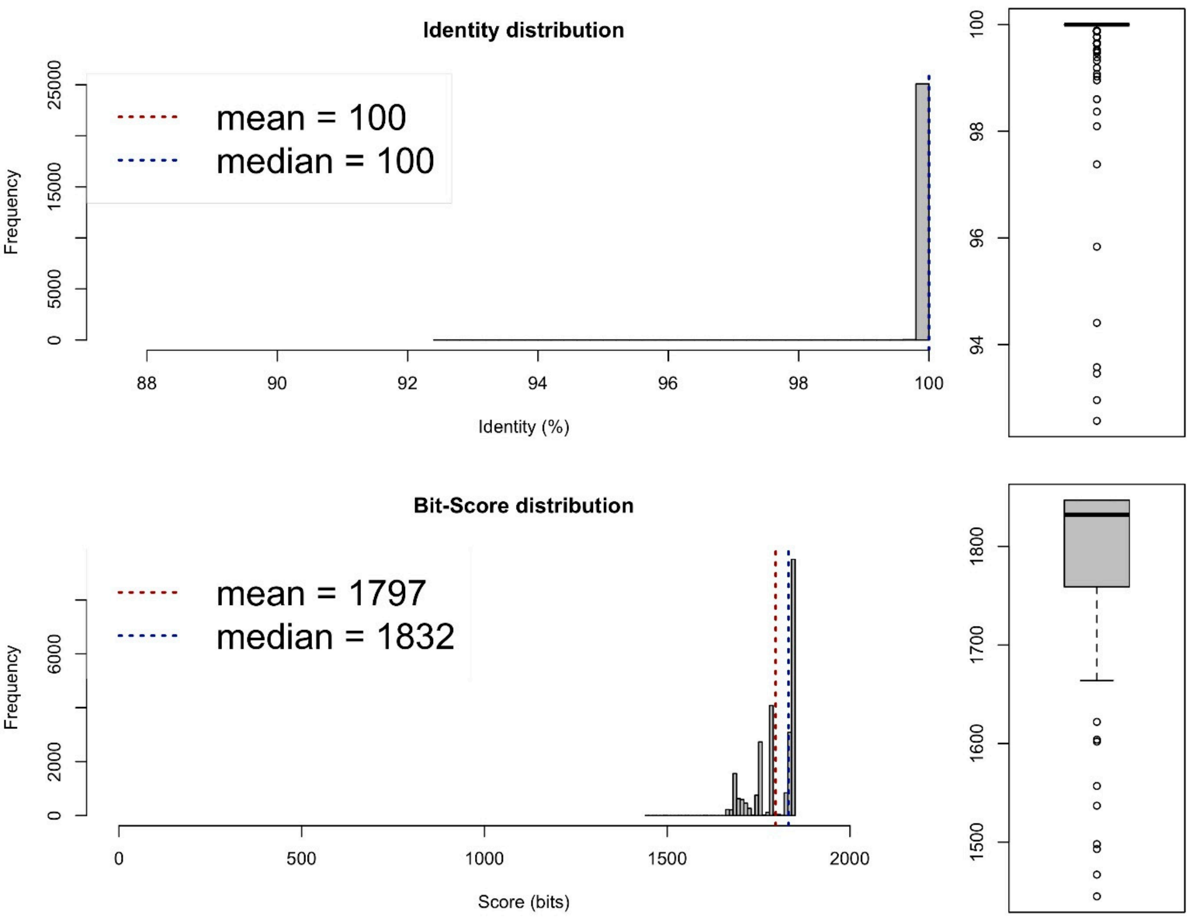

Supplementary Figure S11: The Average Nucleotide Identity (ANI) values between 0× and 1000× amoxicillin genomes of the same species are above 95%. One-way ANI 1: 99.99% (SD: 0.23%), from 25210 fragments. One-way ANI 2: 99.99% (SD: 0.15%), from 25203 fragments. Two-way ANI: 100.00% (SD: 0.10%), from 25145 fragments.

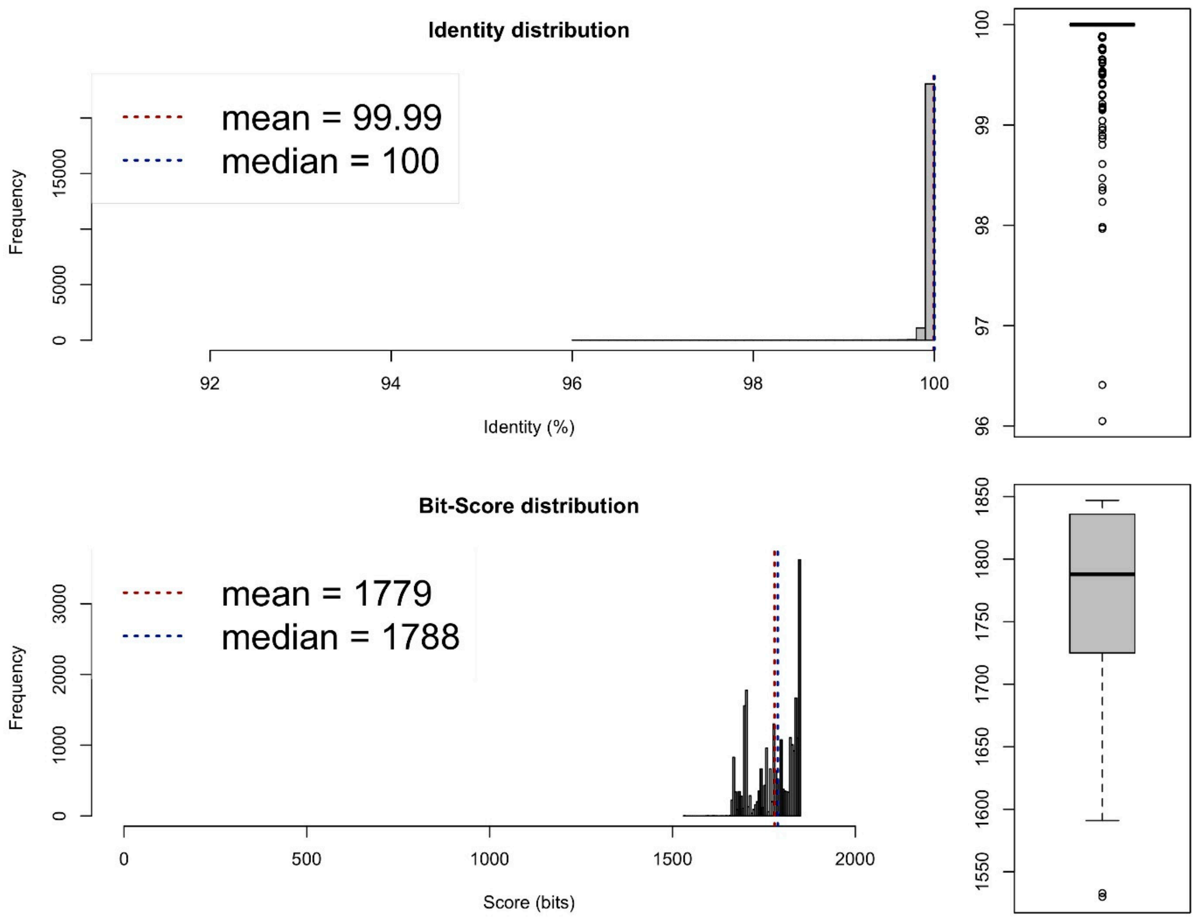

Supplementary Figure S12: The Average Nucleotide Identity (ANI) values between 0× and 1000× cefotaxime genomes of the same species are above 95%. One-way ANI 1: 99.99% (SD: 0.17%), from 24483 fragments. One-way ANI 2: 99.99% (SD: 0.07%), from 24366 fragments. Two-way ANI: 99.99% (SD: 0.07%), from 24307 fragments.
